# Hereditary alpha-tryptasemia and monoclonal mast cell disorders

**DOI:** 10.3389/falgy.2025.1600680

**Published:** 2025-06-19

**Authors:** Yannick Chantran, Michel Arock

**Affiliations:** ^1^ Molecular Platform for the Analysis of cKIT Mutations and Other Gene Defects, ECNM Reference Center, Centre National de Référence des Mastocytoses (CEREMAST), Filière MaRIH, Saint-Antoine Hospital, DMU BioGeMH, AP-HP.Sorbonne University, Paris, France; ^2^ Department of Biological Immunology, Saint-Antoine Hospital, DMU BioGeMH, AP-HP.Sorbonne University, Paris, France; ^3^ Health Environmental Risk Assessment (HERA) Team, UMR261 MERIT, IRD, Inserm 1344, Faculté de Pharmacie, Université Paris Cité, Paris, France; ^4^ Department of Haematology, Pitié-Salpétrière Hospital, DMU BioGeMH, AP-HP.Sorbonne University, Paris, France

**Keywords:** anaphylaxis, tryptase, hereditary alpha-tryptasemia, mastocytosis, monoclonal mast cell disorders

## Abstract

Monoclonal mast cell disorders (mMCD), including systemic mastocytosis, are characterized by the abnormal accumulation of clonal mast cells, often leading to elevated baseline serum tryptase (bST) levels. Thus, bST evaluation is useful for the diagnosis, classification, and management of patients with mMCD. Hereditary alpha-tryptasemia (HαT) is a relatively frequent genetic trait also characterized by elevated bST levels. As compared to the general population, HαT is over-represented among patients with mMCD, and associated with even more frequent and severe mast cell activation symptoms, such as hymenoptera venom-induced anaphylaxis. Although both HαT and mMCD induce increased bST levels, their overlap in laboratory features, and potentially in associated clinical manifestations, have made the diagnostic process of mMCD more accurate but more complicated. In this review, we provide a brief overview of mMCD, the critical role played by bST in their diagnosis, and on HαT as one of the main bST level modifier. Next, we summarize the existing literature regarding the observed association between HαT and mMCD, with particular attention payed to the prevalence of HαT in patients with mMCD, and the clinical manifestations associated with HαT-positive individuals in mMCD. Finally, we discuss the evidence for and against different explanations underlying this association, focusing on HαT's possible influence on diagnostic criteria for mMCD, its potential to act as a modifier of mast cell related symptoms, and its potential role in promoting mast cell proliferation. We conclude with the diagnostic challenges that clinicians face in distinguishing HαT from other mast cell disorders, the role of bST measurement and tryptase genotyping, and propose management strategies for patients with different presentations. This review underlines the value of a comprehensive diagnostic approach to better understand and manage patients with HαT and mMCD.

## 1 Introduction

Monoclonal mast cell disorders (mMCD), which include systemic mastocytosis (SM) and related disorders, are characterized by the abnormal accumulation of clonal mast cells in one or more organs, leading to a wide spectrum of clinical manifestations. The diagnosis of mMCD is partly based on elevated bST levels in the context of characteristic clinical findings and histopathological evidence of mast cell proliferation (1). However, the presence of hereditary alpha-tryptasemia (HαT) in individuals with mMCD complicates the diagnostic work-up, as HαT can elevate bST levels even in the absence of genuine mast cell disease (2). In addition, some studies suggest that individuals with HαT may experience symptoms resembling those of mMCD, including anaphylaxis and other mast cell activation symptoms (2, 3). This overlap presents significant challenges in diagnosis, and a better understanding of the intersection between HαT and mMCD is essential for refining diagnostic criteria and improving patient management.

In this review, we aim to explore in detail the relationship between HαT and mMCD. We will first provide a brief overview of the role of circulating tryptase level masurement as a key marker in the diagnosis, classification, and management of mMCD. Next, we will introduce factors associated with inter-individual bST variability, focusing on HαT, discussing its prevalence, biological implications, and its potential clinical manifestations. The review will then systematically explore the evidence regarding the over-representation of HαT in patients with mMCD, and the clinical presentation in affected individuals. Finally, we will critically examine the possible mechanisms linking HαT to monoclonal mast cell disorders, considering the potential impact of HαT on bST levels, including the potential role of HαT in modifying symptoms such as anaphylaxis, and its potential contribution to mast cell proliferation in the context of mMCD. Through this comprehensive review, we hope to shed light on the complexity of diagnosing and managing patients with both HαT and monoclonal mast cell disorders, providing guidance to clinicians facing these diagnostic dilemmas.

## 2 Tryptase in monoclonal mast cell disorders

Monoclonal mast cell disorders encompass mast cell-related conditions in which investigations provide evidence of clonality. In most cases, this evidence is based on the detection of an activating mutation in the *KIT* gene, most commonly the D816 V mutation in adults, and/or aberrant expression of phenotypic markers on bone marrow mast cells, such as CD2, CD25, or CD30. These monoclonal conditions may or may not meet the diagnostic criteria for cutaneous mastocytosis (CM) or systemic mastocytosis (SM, see Table 1) (1). Regardless of whether the diagnostic criteria for mastocytosis are fulfilled, patients presenting with mMCD may or may not meet the diagnostic criteria for mast cell activation syndrome (MCAS) (1), as presented in Table 2 (4). When patients meet the diagnostic criteria for MCAS, they may be classified as having monoclonal MCAS (MMAS), another monoclonal mast cell activation disorder (MMAD), or monoclonal mast cells of undetermined significance (MMUS) (1, 4, 5).

**Table 1:** 2022 WHO major and minor criteria for systemic mastocytosis and differences with the ICC 2022 criteria.

Criterion Type^a^	Description
Major Criterion	Presence of multifocal, dense infiltrates of mast cells (≥15 mast cells per aggregate) detected in the bone marrow and/or extracutaneous organs.
Minor Criterion	>25% of mast cells in the bone marrow or other extracutaneous organs showing spindle-shaped morphology or atypical forms.
	Detection of any *KIT* activating mutation in the bone marrow, peripheral blood, or other extracutaneous organs.
	Serum total tryptase level >20 ng/mL (not applicable if there is an associated hematologic neoplasm, to be adjusted in the presence of hereditary alpha-tryptasemia (HαT)^b^).
	Expression of CD2 and/or CD25 and/or CD30 in mast cells identified by flow cytometry or immunohistochemistry.

^a^ For the WHO, the diagnostic of systemic mastocytosis requires meeting the major criterion and at least one minor criterion, or at least three minor criteria, while for the ICC the presence of the major criterion is sufficient to diagnose SM, and if the major SM criterion is not fulfilled, three minor SM criteria are required to establish the diagnosis SM.

^b^ Although the optimal way of adjusting for HαT remains to be defined, one way is to divide the basal tryptase level by 1 plus the extra copy numbers (=total copy numbers) of the alpha tryptase gene.

The following table outlines the 2022 WHO-defined major and minor criteria for the diagnosis of systemic mastocytosis. These criteria are used to confirm the presence of the disease and assess its extent.

HαT, hereditary alpha-tryptasemia; ICC, international consensus classification; SM, systemic mastocytosis; WHO, world health organization.

Adapted from Valent et al. (1).

**Table 2:** Diagnostic consensus criteria for MCAS.

A. Typical clinical signs of severe, recurrent (episodic) systemic MCA are present (often in the form of anaphylaxis) (definition of systemic: involving at least 2 organ systems)
B. Involvement of MCs is documented by biochemical studies: preferred marker: increase in serum tryptase level from the individual's baseline to 120% + 2 µg/L^a^
C. Response of symptoms to therapy with MC-stabilizing agents, drugs directed against MC mediator production, or drugs blocking mediator release or the effects of MC-derived mediators^b^

All 3 MCAS criteria (A + B + C) must be fulfilled to call a condition MCAS.

^a^ Other MC-derived biomarkers of MC activation (recommended: 24 h or spot urinary histamine metabolites or PGD2 metabolites) may also be used, but are less specific compared with the increase in serum tryptase level. In addition, to date, no diagnostic thresholds for the increase in these urinary biomarkers have been defined and validated.

^b^ Example: histamine receptor blockers.

MCAS, Mast cell activation syndrome; MCA, mast cell activation; MC, mast cell.

Adapted from Valent et al. (4).

Tryptase measurement is a key tool for the diagnosis and classification of mMCD. One of the three diagnostic criteria for MCAS involves biological evidence of mast cell activation, typically manifested by a significant elevation in acute serum tryptase compared to baseline serum tryptase (bST), see Table 2 (4). In SM, a bST level > 20 µg/L is considered a minor diagnostic criterion (see Table 1) (1). Baseline serum tryptase concentrations are directly related to clonal mast cell burden in those with mMCD. Furthermore, bST levels exceeding 200 µg/L in SM serve as a criterion for high mast cell burden (B-finding) (1), which is critical for classifying SM subtypes and has prognostic and therapeutic implications (6). Finally, bST is a readily accessible and cost-effective tool for monitoring patients with SM, particularly when they are treated by cytoreductive therapy (7, 8).

For these reasons, tryptase is an invaluable tool for the diagnosis and follow-up of mMCD. However, it is known to have limitations in both sensitivity and specificity. Many patients with SM do not meet the bST > 20 µg/L criterion, and many individuals with authentic mast cell activation do not reach the biological MCAS criterion. Additionally, individuals without mMCD may present with elevated bST levels. In recent years, significant efforts have been made to improve our understanding of the determinants of tryptase levels, to refine reference values, to define criteria for significant elevation, and to fine-tune the bST-related criteria to patients' characteristics in the context of mMCD.

## 3 Tryptase, tryptase genetics and hereditary alpha-tryptasemia

In healthy individuals, tryptase is a neutral serine protease almost exclusively produced by mast cells (MCs). Mature active tryptase tetramers are stored in MC granules and released into the extracellular space in large quantities following MC activation, such as during IgE-mediated activation. In contrast, inactive protryptase monomers are continuously released by MCs and diffuse into the bloodstream, where they are detectable even in the absence of MC activation, constituting the bST level (9).

Circulating bST levels remain remarkably stable over time in a given individual, with inter-individual differences being the primary source of variability across a population. Several factors contribute to inter-individual variability in bST levels, including sex, age, tobacco use, body weight, obesity, and other cardiovascular risk factors. Besides mMCD, various conditions—such as kidney failure, active urticaria, and other hematologic malignancies involving the myeloid lineage—have been linked to potentially elevated bST levels (10). In recent years, genetic factors related to tryptase have emerged as one of the main contributors to inter-individual variability in bST levels (11, 12).

The locus encoding secreted tryptases in humans consists of two adjacent genes located on chromosome 16 at position 16p13.3: *TPSAB1*, and *TPSB2*. While *TPSB2* always codes for the β-tryptase isoform, *TPSAB1* can code for either α- or β-tryptase alleles. The adjacent tryptase genes are in linkage disequilibrium, typically co-inherited as haplotypes that follow Mendelian inheritance patterns in familial pedigrees. In the absence of tryptase gene copy number variation (CNV), the two main wild-type haplotypes, αβ and ββ, are nearly evenly distributed in populations of Caucasian descent, with about 25% of individuals without tryptase gene CNV having the ββ:ββ genotype, 50% having the αβ:ββ genotype, and 25% having the αβ:αβ genotype (13).

Several tryptase gene CNVs have been reported since the elucidation of the “canonical” tryptase genetics. Cases of tryptase gene deletions or β-duplications have been documented, though their biological and clinical consequences remain unclear (11). The most studied tryptase gene CNV involves tandem replications of the *TPSAB1* gene, which codes for the α-tryptase allele. This genetic trait, known as HαT, can be accurately detected using digital PCR (dPCR) (2). Duplications, triplications, and even higher numbers of additional copies of the α-tryptase-coding *TPSAB1* gene have been described at the same locus (14, 15).

HαT is a relatively common genetic trait in the general population, with a reported prevalence of 4% to 7% in various control groups, yielding an overall prevalence of 5.2% (150/2,872) (16–21). The only study investigating HαT prevalence in a UK birth cohort also reported a prevalence of 5.2% (17). However, the prevalence of HαT in populations outside of those of European descent remains unknown.

The primary and well-established biological consequence of HαT is its association with elevated bST levels. Tryptase overexpression in HαT may result from a gene-dosage effect—the more copies of the gene present, the higher the bST values—or from an expanded, overactive promoter element co-inherited with additional *TPSAB1* copies (2, 14, 15). In rare cases with very high *TPSAB1* copy numbers, bST levels can exceed 100 µg/L (15). However, in practice, only about 20% of HαT-positive individuals without detectable mMCD have bST levels > 20 µg/L (22).

## 4 HαT and monoclonal mast cell disorders

The reported prevalence, biological and clinical features associated with HαT in different mMCD cohorts are presented in Table 3.

**Table 3:** Main findings associated with HαT in monoclonal mast cell disorders.

Ref.	Lyons et al. (16)	Greiner et al. (18)	Sordi et al. (23)	Polivka et al. (19)	González-de-Olano et al. (21)
Controls					
nature of controls	healthy (*n* = 125) w/o atopic disease (*n* = 398)	w/o hematologic neoplasm (*n* = 180) w/ other myeloid neoplasms (*n* = 720)	na	healthy adults	healthy donor
HαT, % (*n*)	5.6% (7/125) 5.3% (21/398)	4.4% (8/180) 4.6% (33/720)	na	5.7% (15/264)	4.3% (15/346)
mMCD					
nature of mMCD	mastocytosis	Mastocytosis ISM	Mastocytosis or mMCAS	Mastocytosis or mMCAS	mastocytosis
HαT, % (*n*)	12.2% (10/82)	17.2% (31/180) 21.3% (13/61)	13.3% (59/385)	13.5% (79/583)	17.9% (83/464)
Laboratory features					
bST levels	↗	↗	↗	↗	↗
cKIT D816V (%)	↘	=	↘	=	na
MC burden	na	↘	↘	na	=
Clinical features					
MC mediator symptoms (%)	↗	↗	↗	↗	↗
MC mediator symptoms severity	na	↗	na	na	na
Osteopenia/osteoporosis	na	na	↘	=	na
Cutaneous involvement of SM	na	na	↘	↘	na

bST, baseline serum tryptase; HαT, hereditary alpha-tryptasemia; ISM, indolent systemic mastocytosis; MC, mast cell; mMCAS, monoclonal mast-cell activation syndrome; mMCD, monoclonal mast-cell disorders; na, not available; SM, systemic mastocytosis.

### 4.1 Over-representation of HαT in mMCD

The initial association between HαT and mMCD was described in a patient with a *TPSAB1* quintuplication and SM (14). Subsequent cohort studies have consistently found a higher prevalence of HαT in mMCD compared to control groups. Lyons et al. reported a 12.2% (10/82) prevalence of HαT in a U.S. cohort of patients with SM, regardless of baseline serum tryptase (bST) levels or a history of atopy, venom allergy, or anaphylaxis (16). Greiner et al. reported a 17.2% (31/180) prevalence of HαT in patients with mastocytosis, with a prevalence of 21.3% (13/61) in an independent cohort of patients with indolent systemic mastocytosis (ISM) (18). Sordi et al. found a prevalence of HαT of 13.3% (59/385) in an Italian multicenter cohort of patients with mastocytosis or MMAS (23). Polivka et al. reported a prevalence of 13.5% (79/583) in a multicenter French cohort of patients with mastocytosis or MMAS (19). Finally, González-de-Olano et al. reported a prevalence of HαT of 17.9% (83/464) among Spanish patients with mastocytosis (21). Collectively, these studies consistently reported an over-representation of HαT in mMCD and SM as compared to unaffected control groups. Notably, this association was not observed in other myeloid neoplasms and appears to be specific to mast cell-related hematological malignancies (18).

### 4.2 Laboratory features associated with HαT in mMCD

As expected, higher bST levels have been consistently observed in patients with HαT-associated mMCD or mastocytosis. The frequency of the *KIT* D816 V mutation, a hallmark of SM, was found to be similar between HαT-negative and HαT-positive mastocytosis in the German (18) and the French (19) studies, although the D816 V mutation was less frequent in HαT-positive mastocytosis compared to HαT-negative mastocytosis in the Italian cohort (23). However, patients with mastocytosis and HαT appear to present with features of lower mast cell burden, such as lower *KIT* D816 V variant allele frequency (VAF) in blood, bone marrow (BM) aspirates or BM biopsies, lower BM cell infiltration, and fewer BM MC clusters, even within subgroups of SM (18, 19, 23). However, such differences were not observed in the Spanish cohort (21).

### 4.3 Clinical features associated with HαT in mMCD

A consistent finding regarding HαT and mMCD is the higher rate of HαT-positive patients with a history of allergy, particularly anaphylaxis. In the U.S. cohort of SM patients, 90% (9/10) of HαT-positive patients had a history of anaphylaxis, compared to 43% (31/72) of SM patients with the wild-type tryptase genotype (16). Similarly, the German study found hymenoptera venom allergy to be more frequent in HαT-positive patients with mastocytosis as compared to HαT-negative participants with mastocytosis, in both the discovery and validation cohorts (18). In the Italian, French, and Spanish studies, a history of anaphylaxis at inclusion was more common in mastocytosis patients with HαT (19, 21, 23).

Hymenoptera stings are the primary trigger of anaphylaxis in SM, and hymenoptera venom allergy (HVA) was more frequently associated with HαT in mastocytosis patients in the German study (18). However, other studies did not report any significant differences in anaphylaxis triggers when comparing HαT-positive and HαT-negative patients with mMCD (16, 23).

Greiner et al. reported that mast cell activation-related symptoms (including hypotension and anaphylaxis) were more frequent and more severe in HαT-positive mastocytosis patients as compared to HαT-negative patients (18). However, when excluding anaphylaxis from mast cell activation symptoms, other studies did not find any significant association between mast cell activation symptoms or anti-mediator therapy and HαT status in mastocytosis patients (19, 23). In contrast, the Spanish study reported a higher prevalence of headaches and paresthesia, and a lower prevalence of syncope in HαT-positive mastocytosis patients as compared to their wild-type counterparts (21).

Furthermore, some studies have reported a positive correlation between the number of additional copies of *TPSAB1* and the risk of anaphylaxis (21) or the severity of mast cell-related symptoms (18). Regarding other manifestations of mastocytosis, HαT-positive individuals appear less prone to have cutaneous involvement and tend to have a lower prevalence of osteoporosis or osteopenia (19, 23).

Finally, with regard to the outcomes of mastocytosis, overall survival and progression-free survival do not appear to differ significantly between HαT-positive and HαT-negative individuals (23).

## 5 Critical discussion

In this comprehensive discussion, we will critically examine the possible mechanisms linking HαT to mMCD, considering the potential impact of HαT on bST levels, the potential role of HαT in modifying symptoms such as anaphylaxis, and its potential contribution to mast cell proliferation in the context of mMCD.

### 5.1 HαT as a modifier of bST levels

The literature reports an over-representation of HαT in patients with mMCD, suggesting a potential link between these two conditions. In fact, the impact of HαT on bST levels may influence the diagnostic work-up for mMCD, potentially leading to misdiagnosis, but also to facilitated or earlier diagnosis in genuine mMCD.

To date, higher bST levels are the primary biological feature associated with HαT. The discovery of HαT was first based on the study of familial pedigrees exhibiting autosomal dominant hypertryptasemia (3). Circulating tryptase concentrations below 6 µg/L have never been described in HαT, and the vast majority of HαT + individuals present with bST ≥ 8 µg/L, with 90% of HαT + individuals having bST ≥ 11.4 µg/L, and about 20% having bST ≥ 20 µg/L (22). However, it should be noted that most of the data available regarding bST values in HαT come from symptomatic patients with an indication for bST measurement, rather than asymptomatic HαT + individuals from the general population, where bST values may be lower, as suggested by González-de-Olano et al. (21).

Resting MC from HαT-positive individuals exhibit higher intracellular tryptase transcript levels and higher basal secretion rates of pro-tryptase monomers, which can explain the elevated bST levels observed in HαT-positive subjects (2). However, these individuals do not exhibit higher intracellular levels of stored mature tryptase protein nor show different functional properties after activation (2, 3, 24, 25).

At the molecular level, the higher tryptase transcript levels and protein secretion rate can be explained by two main factors. First, bST levels in HαT follow a gene-dosage effect, with higher gene copy numbers being associated with higher bST levels. This effect has been demonstrated with *TPSAB1* triplications compared to duplications (2), and has also been reported in quintuplications or even higher replication numbers (14, 15). However, the number of tryptase gene copies cannot account alone for all the variation in bST levels observed in HαT. This is illustrated by the higher bST levels observed in subjects with HαT associated with tryptase gene deletions in *trans* (26). These individuals present HαT with a normal copy number of alleles, but elevated bST levels. This phenomenon can be explained by an enlarged and overactive promoter element upstream of the duplicated *TPSAB1* allele (15).

Due to the direct impact of HαT on bST levels, HαT-positive individuals may be more likely to undergo diagnostic work-up for SM triggered by unexpectedly high bST levels (18). Furthermore, affected individuals are more likely to meet the bST minor diagnostic criteria for SM, requiring a lower MC burden to reach the critical bST threshold. For these reasons, the HαT-positive SM subset is expected to be enriched for individuals presenting with other indications for bST measurement, such as anaphylaxis, as previously discussed, and with low MC burden SM subtypes.

Indeed, this expected classification shift towards earlier stages of SM and low-burden disease in HαT-positive SM patients compared to HαT-negative SM patients was observed in most cohorts. In the German cohort, the highest prevalence of HαT was observed in patients with indolent SM (ISM) or smoldering SM (SSM), as compared with advanced SM subgroups. Additionally, among patients with TPSAB1 triplications or more, 95% (21/22) of HαT + SM + participants presented with ISM/SSM, compared to 70% (104/149) in HαT-negative SM patients (18). In the French cohort, enrichment of HαT + individuals was statistically significant in the BM mastocytosis (BMM) variant of SM (19.7%, 15/76) and in the ISM variant (11.7%, 31/265) subgroups, but not in other SM variants (19). Similar results were observed in the Italian cohort (23). Furthermore, Greiner et al. showed that within the ISM/SSM variants, HαT-positive individuals had lower *KIT* D816 V allele burden and lower BM MC infiltration, despite having higher bST levels, likely due to the α-tryptase encoding *TPSAB1* copy number gain (18).

Given the impact of α-tryptase encoding *TPSAB1* copy number gain on bST values and the subsequent bias introduced in the bST minor diagnostic criterion for SM, as well as the B-finding criterion regarding bST levels, it was anticipated that these criteria would need to be adapted based on the number of *TPSAB1* additional copies (18). The adaptation of bST-related criteria in SM was swiftly incorporated into consensus diagnostic criteria and the classification of mast cell disorders (1). To what extent this adaptation of bST-related SM diagnostic criteria would dismiss cases that would otherwise have been classified as SM remains unknown. However, recent data suggest that standardization of SM evaluation, including, but not limited to, bST threshold adaptation, could significantly lower HαT prevalence in ISM to the level of the general population (27). Nevertheless, Sordi et al. have reported that applying such an adaptation of bST thresholds in their cohort would only reclassify 7 out of 51 SM patients to MMAS, which would be insufficient to decrease HαT prevalence in SM to the level of the general population (23).

Several algorithms, formulas, and tools were proposed to adapt bST to tryptase genotype: dividing the bST level by (1 + *TPSAB1* extra copy number) (1). In the case of 20 µg/L as a threshold for SM diagnostic, this formula is equivalent to multiplying the cut-off by two (i.e., 40 µg/L) in case of a duplication, by three in case of a triplication (i.e., 60 µg/L), etc. Based on a data-driven model, the BST CALCULATER (for Basal Serum Tryptase Clinical cut-off Assigned by Locus Copy Number of UTR-Linked element and Associated *TPSAB1* Encoded Replication) propose upper limits for predicted bST levels based upon replication number (15). Using this model, the predicted 99.5th percentiles for *TPSAB1* duplication and triplication yield 36 µg/L and 62 µg/L, respectively, which is very close to the bST cut-offs adapted by the first formula (40 µg/L and 60 µg/L, respectively). Other formulas are available to interpret acute tryptase elevation, such as the Valent's formula (aST ≥ 1.2*bST + 2) (1), or the Mateja's formula (aST ≥ 1.685*bST) computed online by the Total Rise In Peripheral Tryptase After Systemic Event (TRIPTASE) Calculator (28). Additionally, we and others (29) developed online tools to provide with age- and sex-specific bST reference values (see, for instance, https://sfa.lesallergies.fr/tryptase_calculator). Regarding the adaptation of bST thresholds in mMCD, the best formula in cases of HαT remains open for debate, and further refinement could increase not only specificity but also sensitivity by lowering bST thresholds in subjects without *TPSAB1* additional copies (1, 28, 30).

### 5.2 HαT as a symptom modifier in monoclonal mast cell disorders

One plausible hypothesis to explain the observed association between HαT and mMCD is that HαT might influence the severity of clinical symptoms in individuals with mMCD, such as anaphylaxis and other mast cell activation disorders. HαT might exacerbate these symptoms due to its effects on mast cell activation or might lead to a heightened sensitivity to mast cell degranulation. This could explain why patients with HαT and mMCD present with more severe or earlier manifestations of mast cell activation, potentially influencing clinical decision-making. In some instances, clinicians may be biased toward diagnosing mMCD in HαT patients with elevated bST levels, leading to earlier recognition and treatment. This hypothesis suggests that HαT might not just modify the clinical presentation but could also serve as a contributing factor to earlier diagnosis, which could have therapeutic benefits.

Le et al. provided a pathophysiological basis for linking tryptase genotypes with potential clinical phenotypes. Individuals expressing α-tryptase due to normal or additional *TPSAB1* copies produce α/β-tryptase heterotetramers, which possess unique properties compared to conventional β-tryptase and enzymatically inactive α-tryptase homotetramers (31). Indeed, these heterotetramers can promote vibration-elicited urticaria, a common symptom in HαT individuals, through preactivation of the mast cell mechanosensory receptor EMR2. Additionally, α/β-tryptase heterotetramers can activate the protease-activated receptor-2 (PAR2), which may contribute to mast cell activation-related manifestations such as smooth muscle constriction, epithelial activation, and increased vascular permeability (16, 31).

Apart from mMCD, HαT has been associated with a variety of clinical manifestations, as reported in seminal papers describing HαT in familial pedigrees (2, 3). Affected individuals exhibited a constellation of symptoms when compared to unaffected family members, many of which resembling those associated with MCAS (32). These symptoms were initially categorized into five groups: (i) cutaneous symptoms (flush, urticaria, angioedema, pruritus); (ii) connective tissue manifestations (joint hypermobility, retained primary dentition, congenital skeletal abnormalities); (iii) atopy (hymenoptera venom anaphylaxis, eczema, asthma, environmental allergies, food allergies, drug allergies); (iv) gastrointestinal symptoms (inflammatory bowel syndrome, episodic diarrhea, abdominal pain, gastroesophageal reflux disease); and (v) neuropsychiatric manifestations (dysautonomia, postural orthostatic tachycardic syndrome, chronic pain, fibromyalgia, vulvodynia, sleep disturbances, anxiety, and depression) (3). A higher prevalence of systemic venom reactions, cutaneous flush or pruritus, inflammatory bowel syndrome, dysautonomia, and retained primary dentition was first confirmed in HαT + individuals from an unselected cohort (2). However, since HαT affects 4%–6% of the western population, full clinical penetrance of this frequent genetic trait is unlikely, unless conferring a strong reproductive advantage. Whether HαT induces clinical manifestations in the general population remains a matter of vivid debate, particularly since the first clinical description was not confirmed by a more recent study (20, 33, 34). Recently, HαT has been linked to cold-induced urticaria and cold-induced anaphylaxis, further reigniting the debate about the nature and penetrance of HαT symptoms outside of mMCD (35).

In the context of mMCD, the association between HαT and anaphylaxis is a consistent finding across studies, suggesting that HαT may serve as a genuine symptom modifier. However, as presented earlier, it is important to note that among SM cohorts, HαT has been broadly associated with a shift toward low-MC burden SM variants, such as ISM and/or BMM, and patients without SM typical skin lesions, but rather with history of HVA as a red flag leading to diagnosis (18, 19, 23). Arguably, a BMM/ISM patient with an unknown mMCD who presents with severe HVA and elevated bST levels—at least partly due to HαT—would be more likely to undergo investigation for and diagnosis of SM compared to the same patient without HαT. Thus, it is conceivable that the enrichment in the HαT-positive subset observed in SM cohorts, specifically regarding anaphylaxis, may be partly due to selection biases rather than a genuine pathophysiological relationship between HαT and the risk of anaphylaxis. Importantly, in the Spanish cohort (21), while a history of anaphylaxis at SM diagnosis was more frequent in HαT-positive participants, there was no higher incidence of new anaphylactic events during follow-up among HαT-positive SM patients as compared to HαT-negative SM patients, which advocates against HαT as a symptom modifier in mMCD.

### 5.3 HαT as a potential causal factor in monoclonal mast cell disorders

The higher prevalence of HαT in SM and the germline nature of HαT raise the question of whether HαT could be considered a risk factor for the development of mMCD or whether the two conditions are merely coincidentally associated. One possibility is that the increased mast cell burden seen in HαT could contribute to the abnormal accumulation of mast cells in organs and tissues, a hallmark of mMCD. HαT might thus act as a potential causal factor in the development of monoclonal mast cell diseases, possibly by driving the expansion of mast cells or promoting mast cell activation in individuals already predisposed to mast cell disorders. Indeed, HαT-positive individuals with non-monoclonal MCAS display a higher frequency of unclustered BM MC, which are often larger, hypogranulated, and sometimes spindle-shaped, and may be associated with eosinophilia (3, 36). Furthermore, HαT-positive individuals with gastrointestinal symptoms exhibit higher mast cell numbers in the intestine, often associated with class-switched B-cells (37, 38). These observations suggest that HαT could, in some instances, facilitate the accumulation and/or activation of normal or abnormal MC.

Nevertheless, a potential causal relationship between a CNV of an enzymatic effector protein produced by MC, namely tryptase, and a much more upstream signaling protein, namely the KIT receptor, remains elusive. Moreover, in many cases, the *KIT* mutation in mMCD is found with multilineage involvement, suggesting early acquisition during hematopoiesis, a stage where tryptase is unlikely to be involved. Several potential mechanisms, such as other genetic mutations, epigenetic factors, or changes in mast cell signaling pathways, could however explain a causal link between HαT and mMCD. Further research into these mechanisms is essential to better understand how HαT could contribute to mast cell proliferation and disease development.

It is also noteworthy to observe HαT prevalence in mMCD subsets that are less likely to be diagnosed based on anaphylactic reactions or MCAS, or based on tryptase levels. For example, skin lesions are the chief complaint in cases of pure CM. In CM, various studies report a HαT prevalence of 9.1%, 12.5%, 20.7%, and 23% (18, 19, 21, 23). Overall, the HαT prevalence in pure CM appears to reach 16.2%, a feature similar to that found in ISM and higher than expected in the general population. In addition, HαT was recently found to affect 19.4% (6/31) of children with solitary mastocytoma (39), where extracutaneous mediator-related symptoms can happen, but are not a chief complaint (40).

A similar rationale applies to advanced SM, where the primary complaints leading to diagnosis are related to organ impairment due to mast cell infiltration, rather than MCAS or anaphylaxis. In advanced SM, virtually all patients exhibit very high bST levels due to huge mast cell burden, far surpassing the 20 µg/L threshold. In this context, the presence of HαT should not critically bias the fulfilment of diagnostic criteria. In advanced SM, different cohorts report HαT prevalence ranging from 10.6% to 13.2% (18, 19, 21, 23), with an overall prevalence of 11.7%, which is again similar to the prevalence in ISM and higher than expected in the general population, as nicely discussed by Polivka et al. (19).

## 6 Conclusion and perspectives

The relationship between HαT and mMCD poses considerable challenges for clinicians, primarily due to the shared laboratory findings of both conditions, and possibly due to common clinical features. This overlap complicates the diagnostic process, as elevated bST levels—traditionally a key diagnostic marker for mMCD—can also be observed in HαT patients without genuine mast cell proliferation.

Research continues to unravel the complex interplay between HαT and mMCD. However, insofar HαT undoubtedly acts as a biological modifier of bST levels, it has become clear that the bST thresholds that are used as a minor diagnostic criteria and as a B-finding in SM should be adapted to tryptase genotype (1, 15). Accordingly, patients with bST > 20 µg/L and/or high clinical suspicion of SM, e.g., based on the presence of *urticaria pigmentosa*, severe HVA, or high REMA score, should undergo tryptase genotype along with BM evaluations as part of diagnostic work-up.

One of the primary diagnostic challenges lies in distinguishing between elevated tryptase levels due to HαT and those indicative of genuine mast cell proliferation, among individuals with low clinical suspicion of mMCD, e.g., the fortuitous discovery of an elevated bST after an anaphylactic event not suggestive of underlying mMCD. In such cases, considering that only elevated bST levels would have suggested mMCD, while the full clinical presentation, including the patient's history, symptoms, and other diagnostic criteria, would not have raised any suspicion of mMCD, we propose to limit investigations to peripheral blood (PB) as a first step. We recommend that symptomatic patients presenting with bST ≥ 8 µg/L should first be tested for HαT and PB *KIT* D816 V. We suggest carrying on BM investigations in those with positive PB *KIT* D816 V, bST levels exceeding genotype-corrected bST, or presenting with clinical manifestations highly suggestive of SM.

Given the complexities of diagnosing HαT and mMCD, it is essential for clinicians to adopt a nuanced approach not only to diagnosis, but also for patient's management. One pressing issue concerns the management of subjects with HαT, with or without associated mMCD, regarding their risk of severe anaphylaxis. To which patients, if any, epinephrine auto-injectors should be prescribed? Since HαT is a non-modifiable potential risk factor of severity, should HαT-positive subjects undergo lifelong immunotherapy, if indicated? Further studies are needed to address these questions. However, given the very low prevalence of severe anaphylaxis in the general population, which contains about 5% of HαT-positive individuals, the additional risk of severe anaphylaxis attributable to HαT should be very low.

Whether HαT induces clinical manifestations in the general population, and whether the observed association of HαT and SM is true, remains a matter of debate. Large prospective population-based study are needed to provide insights regarding the relationship between HαT, other biological variables, and clinical manifestations. However, population-based studies which include thorough clinical investigations and blood sampling are often limited to few thousands participants, at best. The association between HαT and frequent clinical complaints, such as atopic diseases or gastrointestinal symptoms, could be investigated by such study design. However, such studies in the general population are unlikely to be informative regarding very rare clinical events, such as anaphylaxis or mMCD. In the future, innovative *in vitro* or animal models involving tryptase genes could help to elucidate the potential pathophysiological relationship between HαT, mMCD, and its manifestations.

In conclusion, the explanation of the observed association between HαT and mMCD is complex, probably multifactorial, and mainly unresolved. Without a doubt, HαT-status must be taken into account for the diagnostic evaluation for mMCD, because of its impact on bST levels. While HαT may also modify the clinical presentation of mMCD, it is still unclear whether HαT acts as a direct causal factor in mast cell disease pathogenesis. Further research into the molecular mechanisms underlying this association, as well as critical reflexion regarding selection biases in clinical studies, are crucial for developing better diagnostic strategies and more effective management for patients with both conditions.

## References

[1] Valent P Akin C Hartmann K Alvarez-Twose I Brockow K Hermine O Updated diagnostic criteria and classification of mast cell disorders: a consensus proposal. HemaSphere. (2021) 5(11):e646. 10.1097/HS9.0000000000000646 34901755 PMC8659997

[2] Lyons JJ Yu X Hughes JD Le QT Jamil A Bai Y Elevated basal serum tryptase identifies a multisystem disorder associated with increased TPSAB1 copy number. Nat Genet. (2016) 48(12):1564–9. 10.1038/ng.3696 27749843 PMC5397297

[3] Lyons JJ Sun G Stone KD Nelson C Wisch L O'brien M Mendelian Inheritance of elevated serum tryptase associated with atopy and connective tissue abnormalities. J Allergy Clin Immunol. (2014) 133(5):1471–4. 10.1016/j.jaci.2013.11.039 24472624 PMC4016972

[4] Valent P Hartmann K Bonadonna P Gülen T Brockow K Alvarez-Twose I Global classification of mast cell activation disorders: an ICD-10-CM–adjusted proposal of the ECNM-AIM consortium. J Allergy Clin Immunol Pract. (2022) 10(8):1941–50. 10.1016/j.jaip.2022.05.007 35623575

[5] Ballul T Sabato V Valent P Hermine O Lortholary O Rossignol J Characterization of patients with clonal mast cells in the bone marrow with clinical significance not otherwise specified. EClinicalMedicine. (2025) 80:103043. 10.1016/j.eclinm.2024.103043 39877259 PMC11773267

[6] Lim KH Tefferi A Lasho TL Finke C Patnaik M Butterfield JH Systemic mastocytosis in 342 consecutive adults: survival studies and prognostic factors. Blood. (2009) 113(23):5727–36. 10.1182/blood-2009-02-205237 19363219

[7] Erben P Schwaab J Metzgeroth G Horny H-P Jawhar M Sotlar K The KIT D816V expressed allele burden for diagnosis and disease monitoring of systemic mastocytosis. Ann Hematol. (2014) 93(1):81–8. 10.1007/s00277-013-1964-1 24281161

[8] Gotlib J Reiter A Radia DH Deininger MW George TI Panse J Efficacy and safety of avapritinib in advanced systemic mastocytosis: interim analysis of the phase 2 PATHFINDER trial. Nat Med. (2021) 27(12):2192–9. 10.1038/s41591-021-01539-8 34873345 PMC8674139

[9] Khoury P Lyons JJ. Mast cell activation in the context of elevated basal serum tryptase: genetics and presentations. Curr Allergy Asthma Rep. (2019) 19(12):55. 10.1007/s11882-019-0887-x 31776770

[10] Michel M Klingebiel C Vitte J. Tryptase in type I hypersensitivity. Ann Allergy Asthma Immunol. (2023) 130(2):169–77. 10.1016/j.anai.2022.08.996 36084866

[11] Wu R Lyons JJ. Hereditary alpha-tryptasemia: a commonly inherited modifier of anaphylaxis. Curr Allergy Asthma Rep. (2021) 21(5):33. 10.1007/s11882-021-01010-1 33970354

[12] Chantran Y Choi S Roda C Nicaise-Roland P de Chaisemartin L Chollet-Martin S Higher levels of basal serum tryptase are associated with sensitization, FeNO, allergic morbidity, and lower control of allergic asthma in teenagers from the PARIS birth cohort. Allergy. (2024) 80:584. 10.1111/all.16284 39155860 PMC11804298

[13] Trivedi NN Tamraz B Chu C Kwok PY Caughey GH. Human subjects are protected from mast cell tryptase deficiency despite frequent inheritance of loss-of-function mutations. J Allergy Clin Immunol. (2009) 124(5):1099–1105.e1–4. 10.1016/j.jaci.2009.07.026 19748655 PMC2783561

[14] Sabato V Chovanec J Faber M Milner JD Ebo D Lyons JJ. First identification of an inherited TPSAB1 quintuplication in a patient with clonal mast cell disease. J Clin Immunol. (2018) 38(4):457–9. 10.1007/s10875-018-0506-y 29748908

[15] Chovanec J Tunc I Hughes J Halstead J Mateja A Liu Y Genetically determining individualized clinical reference ranges for the biomarker tryptase can limit unnecessary procedures and unmask myeloid neoplasms. Blood Adv. (2023) 7:1796–819. 10.1182/bloodadvances.2022007936 36170795 PMC10164828

[16] Lyons JJ Chovanec J O’Connell MP Liu Y Šelb J Zanotti R Heritable risk for severe anaphylaxis associated with increased α-tryptase–encoding germline copy number at TPSAB1. J Allergy Clin Immunol. (2021) 147(2):622–32. 10.1016/j.jaci.2020.06.035 32717252

[17] Robey RC Wilcock A Bonin H Beaman G Myers B Grattan C Hereditary alpha-tryptasemia: UK prevalence and variability in disease expression. J Allergy Clin Immuno Pract. (2020) 8(10):3549–56. 10.1016/j.jaip.2020.05.057 32553831

[18] Greiner G Sprinzl B Górska A Ratzinger F Gurbisz M Witzeneder N Hereditary α tryptasemia is a valid genetic biomarker for severe mediator-related symptoms in mastocytosis. Blood. (2021) 137(2):238–47. 10.1182/blood.2020006157 32777817 PMC7116780

[19] Polivka L Madrange M Bulai-Livideanu C Barete S Ballul T Neuraz A Pathophysiologic implications of elevated prevalence of hereditary alpha-tryptasemia in all mastocytosis subtypes. J Allergy Clin Immunol:(2024) 153:349–53. 10.1016/j.jaci.2023.08.015 37633651

[20] Chollet MB Akin C. Hereditary alpha tryptasemia is not associated with specific clinical phenotypes. J Allergy Clin Immunol. (2022) 149(2):728–735.e2. 10.1016/j.jaci.2021.06.017 34174297

[21] González-de-Olano D Navarro-Navarro P Muñoz-González JI Sánchez-Muñoz L Henriques A de-Andrés-Martín A Clinical impact of the *TPSAB1* genotype in mast cell diseases: a REMA study in a cohort of 959 individuals. Allergy:79:711–23. 10.1111/all.15911 37818990

[22] Giannetti MP Weller E Bormans C Novak P Hamilton MJ Castells M. Hereditary alpha-tryptasemia in 101 patients with mast cell activation–related symptomatology including anaphylaxis. Ann Allergy Asthma Immunol. (2021) 126(6):655–60. 10.1016/j.anai.2021.01.016 33465452

[23] Sordi B Vanderwert F Crupi F Gesullo F Zanotti R Bonadonna P Disease correlates and clinical relevance of hereditary α-tryptasemia in patients with systemic mastocytosis. J Allergy Clin Immunol:151:485–93. 10.1016/j.jaci.2022.09.038 36309122

[24] Giannetti MP Godwin G Weller E Butterfield JH Castells M. Differential mast cell mediators in systemic mastocytosis and hereditary α-tryptasemia. J Allergy Clin Immunol. (2022) 150(5):1225–7. 10.1016/j.jaci.2022.04.025 35550148

[25] Glover SC Nouri M Tuna KM Mendoza Alvarez LB Ryan LK Shirley JF Lipidomic analysis of urinary exosomes from hereditary α-tryptasemia patients and healthy volunteers. FASEB Bioadv. (2019) 1(10):624–38. 10.1096/fba.2019-00030 31803861 PMC6892164

[26] Glover SC Carlyle A Lyons JJ. Hereditary alpha-tryptasemia despite normal tryptase-encoding gene copy number owing to copy number loss in trans. Ann Allergy Asthma Immunol. (2022) 128(4):460–1. 10.1016/j.anai.2021.12.008 34896311 PMC8977281

[27] McMurray JC Pacheco CS Schornack BJ Sun X Brunader JA Scott AE Standardized indolent systemic mastocytosis evaluations across a health care system: implications for screening accuracy. Blood. (2024) 144(4):408–19. 10.1182/blood.2023023347 38635793 PMC11418066

[28] Mateja A Wang Q Chovanec J Kim J Wilson KJ Schwartz LB Defining baseline variability of serum tryptase levels improves accuracy in identifying anaphylaxis. J Allergy Clin Immunol. (2022) 149(3):1010–1017.e10. 10.1016/j.jaci.2021.08.007 34425177 PMC9126046

[29] Puel M Rossignol J Devin C Madrange M Diep A Roland PN Redefining tryptase norms in the pediatric population reveals sex-based differences: clinical implications. Allergy:80:335. 10.1111/all.16365 39425617 PMC11724225

[30] Valent P Hoermann G Bonadonna P Hartmann K Sperr WR Broesby-Olsen S The normal range of baseline tryptase should be 1 to 15 ng/mL and covers healthy individuals with HαT. J Allergy Clin Immunol Pract. (2023) 11(10):3010–20. 10.1016/j.jaip.2023.08.008 37572755 PMC11869997

[31] Le QT Lyons JJ Naranjo AN Olivera A Lazarus RA Metcalfe DD Impact of naturally forming human α/β-tryptase heterotetramers in the pathogenesis of hereditary α-tryptasemia. J Exp Med. (2019) 216(10):2348–61. 10.1084/jem.20190701 31337736 PMC6780998

[32] Gülen T Akin C Bonadonna P Siebenhaar F Broesby-Olsen S Brockow K Selecting the right criteria and proper classification to diagnose mast cell activation syndromes: a critical review. J Allergy Clin Immunol Pract. (2021) 9(11):3918–28. 10.1016/j.jaip.2021.06.011 34166845

[33] Lyons JJ. On the complexities of tryptase genetics and impact on clinical phenotypes. J Allergy Clin Immunol. (2021) 148(5):1342–3. 10.1016/j.jaci.2021.08.011 34489125

[34] Akin C Chollet MB. Reply. J Allergy Clin Immunol. (2021) 148(5):1343–4. 10.1016/j.jaci.2021.08.012 34489124

[35] Bizjak M Korošec P Košnik M Šelb J Bidovec-Stojkovič U Svetina M Cold-induced anaphylaxis: new insights into clinical and genetic characteristics. Front Immunol. (2025) 16:1558284. 10.3389/fimmu.2025.1558284 40061949 PMC11885499

[36] Giannetti MP Akin C Hufdhi R Hamilton MJ Weller E Van Anrooij B Patients with mast cell activation symptoms and elevated baseline serum tryptase level have unique bone marrow morphology. J Allergy Clin Immunol. (2021) 147(4):1497–1501.e1. 10.1016/j.jaci.2020.11.017 33248113

[37] Konnikova L Robinson TO Owings AH Shirley JF Davis E Tang Y Small intestinal immunopathology and GI-associated antibody formation in hereditary alpha-tryptasemia. J Allergy Clin Immunol. (2021) 148(3):813–821.e7. 10.1016/j.jaci.2021.04.004 33865872 PMC9017395

[38] Hamilton MJ Zhao M Giannetti MP Weller E Hufdhi R Novak P Distinct small intestine mast cell histologic changes in patients with hereditary alpha-tryptasemia and mast cell activation syndrome. Am J Surg Pathol. (2021) 45(7):997–1004. 10.1097/PAS.0000000000001676 33481382 PMC8192345

[39] Madrange M Rossignol J Devin C Bekel L Bellon N Welfringer-Morin A A high prevalence of hereditary alpha-tryptasemia in pediatric mastocytoma. Allergy. (2024) 79(11):3129–32. 10.1111/all.16185 38850228

[40] Brockow K Plata-Nazar K Lange M Nedoszytko B Niedoszytko M Valent P. Mediator-Related symptoms and anaphylaxis in children with mastocytosis. Int J Mol Sci. 2021;22(5):2684. 10.3390/ijms22052684 33799959 PMC7962052

